# Peptidomimetic Oligomers Targeting Membrane Phosphatidylserine Exhibit Broad Antiviral Activity

**DOI:** 10.1021/acsinfecdis.3c00063

**Published:** 2023-08-02

**Authors:** Patrick M. Tate, Vincent Mastrodomenico, Christina Cunha, Joshua McClure, Annelise E. Barron, Gill Diamond, Bryan C. Mounce, Kent Kirshenbaum

**Affiliations:** † Department of Chemistry, New York University, New York, New York 10003, United States; ‡ Department of Microbiology and Immunology, Loyola University Chicago Medical Center, Maywood, Illinois 60130, United States; § Maxwell Biosciences, Austin, Texas 78738, United States; ∥ Department of Bioengineering, Stanford University, Stanford, California 94305, United States; ⊥ Department of Oral Immunology and Infectious Diseases, University of Louisville School of Dentistry, Louisville, Kentucky 40292, United States

**Keywords:** peptoid, biomimicry, enveloped virus, antimicrobial peptide

## Abstract

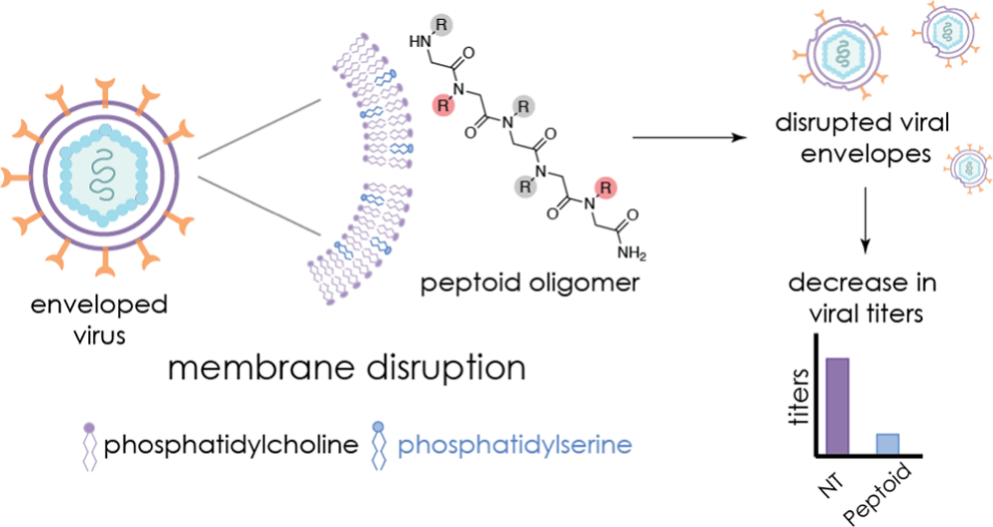

The development of durable new antiviral therapies is challenging, as viruses can evolve rapidly to establish resistance and attenuate therapeutic efficacy. New compounds that selectively target conserved viral features are attractive therapeutic candidates, particularly for combating newly emergent viral threats. The innate immune system features a sustained capability to combat pathogens through production of antimicrobial peptides (AMPs); however, these AMPs have shortcomings that can preclude clinical use. The essential functional features of AMPs have been recapitulated by peptidomimetic oligomers, yielding effective antibacterial and antifungal agents. Here, we show that a family of AMP mimetics, called peptoids, exhibit direct antiviral activity against an array of enveloped viruses, including the key human pathogens Zika, Rift Valley fever, and chikungunya viruses. These data suggest that the activities of peptoids include engagement and disruption of viral membrane constituents. To investigate how these peptoids target lipid membranes, we used liposome leakage assays to measure membrane disruption. We found that liposomes containing phosphatidylserine (PS) were markedly sensitive to peptoid treatment; in contrast, liposomes formed exclusively with phosphatidylcholine (PC) showed no sensitivity. In addition, chikungunya virus containing elevated envelope PS was more susceptible to peptoid-mediated inactivation. These results indicate that peptoids mimicking the physicochemical characteristics of AMPs act through a membrane-specific mechanism, most likely through preferential interactions with PS. We provide the first evidence for the engagement of distinct viral envelope lipid constituents, establishing an avenue for specificity that may enable the development of a new family of therapeutics capable of averting the rapid development of resistance.

The pharmacological limitations of current antivirals underscore the importance of identifying molecules with novel mechanisms of action that are capable of addressing diverse emerging viral threats. Antiviral design has typically followed one of two paths: directly targeting the viral pathogen or targeting the host factors. As viruses require multiple stages in their replicative life cycle, each step can be considered as a target for antiviral drug development. However, viral polymerases exhibit low replication fidelity, enabling them to overcome antiviral treatment modalities through rapid generation of resistance mutations.^1^ Resistance to antivirals has been widely observed for human immunodeficiency virus (HIV), hepatitis B virus, influenza virus, and many other RNA viruses, emphasizing the challenge for developing novel, long-lasting treatments.^2,3^ In addition, host cell dependence is a requirement for virus replication, yet targeting host factors can result in cytotoxicity and severe side effects in patients.^4^ The recent COVID-19 pandemic highlights the aforementioned challenges of designing therapies against viruses, especially considering the prospect of preparing for the next virus outbreak of unknown origin. Throughout the recent pandemic, variants of SARS-CoV-2 emerged that posed a public health risk due to higher transmissibility and disease severity. Ideally, the design of new antivirals would enable the retention of efficacy against emerging variants of concern, even as they undergo extensive alteration of their protein sequences. The emergence of COVID variants is indicative of the general challenges in establishing robust treatment regimens for viruses of pandemic potential. One avenue for addressing these challenges is to identify therapeutic targets that are conserved and specific to the virus and are non-toxic to host cells.

Viruses that include a lipid membrane surrounding the protein capsid and viral genome are categorized as enveloped viruses. Viruses lacking such a membrane are categorized as non-enveloped. Notably, the innate immune system can target pathogen membranes by constitutively expressing short antimicrobial peptides (AMPs). The physicochemical characteristics of AMPs allow them to exert direct antimicrobial activity at the pathogen membrane surface.^5^ Over 3000 AMPs are synthesized by a wide variety of different organisms, and more than 2000 are active against viruses.^6^

Mechanistic studies have extensively investigated how AMPs function against bacteria, but studies of AMPs as antivirals have received less attention. α- and β-defensins have been suggested to play a key role in the innate immune response against RNA and DNA viruses through a variety of mechanisms.^7^ AMP inhibition of respiratory syncytial virus, vaccinia virus, influenza A virus, Zika virus (ZIKV), HIV, and hepatitis C virus by envelope pore formation or membrane disruption have been observed.^8−13^ Additionally, AMPs can cause viral particle aggregation, leading to reduced infectivity, as seen with Venezuelan equine encephalitis virus (VEEV).^14^ Minimal investigation has been conducted on the interactions between AMPs and specific lipid constituents at the membrane interface of enveloped viruses, although there are likely implications for virus particle stability and infectivity. The outstanding questions regarding the role of AMPs as antiviral agents can be addressed by studying how these peptides exert mechanisms of action to inactivate viral pathogens.

Despite the large number and broad activities of AMPs, they are rarely utilized in the clinic to combat human infectious disease. Peptide therapeutics often exhibit poor bioavailability, unwanted immunogenicity, and can be costly to synthesize—all of which limit their clinical use. Peptoids, or *N*-alkylated glycine oligomers, are sequence-specific peptidomimetic compounds with side chains located on backbone amide nitrogens, rather than on the backbone α-carbon (as found in peptides). Relative to peptides, peptoids have greater membrane permeability and are not prone to proteolytic degradation.^15,16^ Advantageously, extensive chemical diversity of side chain groups is readily accessed by selection from a broad range of amine “submonomer” reagents. The solid-phase synthesis of peptoids is modular, rapid, economical, and amenable to scale-up.^17^ Natural α-helical AMPs have been extensively studied as drug candidates; however their aforementioned shortcomings clearly indicate the potential advantages of synthetic biomimetic agents, such as peptoids.

A variety of peptoid sequences incorporating cationic and hydrophobic side-chain groups have been reported to show analogous structural and functional characteristics as AMPs.^18^ The three linear peptoids used in this study, designated as MXB004, MXB005, and MXB009, were identified by the Barron lab from a library of bioactive peptoids that displayed potent antibacterial activity.^19^ Previous work has focused on identifying the antibacterial and antifungal activity of these peptoids. Recent preliminary studies conducted by Diamond et al. have shown that MXB004, MXB005, and MXB009 also exhibit potent in vitro antiviral activity against HSV-1 and SARS-CoV-2.^20^ Cryo-EM images revealed extensive viral envelope disruption, suggesting that these peptoids act via membrane-based mechanisms to inactivate enveloped viruses, but further work is needed to understand how peptoids engage envelope constituents.

Due to the AMP-like characteristics of antimicrobial peptoids described above, we were interested in investigating the role that the viral envelope may play in peptoid-mediated antiviral activity. The physical and chemical differences between the host and viral membranes make viral envelopes attractive targets for new therapeutics. Although enveloped viruses acquire their lipids from the host cell, the composition of viral membranes differs significantly from that of the host.^21−23^ Viral lipid heterogeneity is a growing research focus, and ongoing lipidomic studies of viruses are highlighting the importance of diverse lipids in virus infection.^24^ The lipid composition of viral envelopes includes phosphatidylcholine (PC), phosphatidic acid, phosphatidylglycerol, sphingolipids, phosphatidylethanolamine, and phosphatidylserine (PS) (among others) in concentrations that are distinct from host cells.^23,25^ PS plays important physiological roles in eukaryotic cells where it can be used as a signal for apoptosis to induce subsequent phagocytosis.^26^ PS exposure on the outer membrane of eukaryotic cells is tightly regulated by flippases; however, during apoptosis, scramblases can induce the movement of PS from the inner to the outer membrane leaflet.^27,28^ Notably, viruses take advantage of PS-mediated uptake to facilitate viral entry.^29−31^ Viruses typically have an increased PS content presented on their outer surface relative to host cell membranes in order to engage with PS-mediated cellular entry pathways. These differences make PS within the envelope a specific and attractive target when designing therapeutics against enveloped viruses. Previous studies have evaluated oligomeric compounds targeting membrane lipid constituents as anti-cancer therapeutics.^32^ Preferential binding to negatively charged phospholipids, including PS, was observed for an oligomer incorporating peptoid monomers.^33^ These results suggest the potential for targeting phospholipids with peptoid oligomer anti-infective agents.

We were interested in studying the antiviral effects of antimicrobial peptoids against four distinct viral pathogens that currently have no available treatment or vaccine options. ZIKV, Rift Valley Fever virus (RVFV), chikungunya virus (CHIKV), and coxsackie B3 virus (CVB3) represent a set of viruses with unique genomes, viral entry pathways, and varying pathologies within a host. The first virus we explored was ZIKV, an enveloped *Flavivirus*, which has a single-stranded RNA genome. ZIKV can result in congenital abnormalities and microcephaly of fetuses, along with the development of Guillain-Barre syndrome in patients.^34^ RVFV, a tri-segmented virus belonging to the *Phlebovirus* genus, was chosen as the second model virus as it can cause severe illness and mortality in livestock and has approximately 10% mortality rate in human patients.^35,36^ The third enveloped virus used in this study was CHIKV, an *Alphavirus* that can cause fever, rash, and disabling arthritis.^37^ Finally, CVB3 was used as a model non-enveloped virus for the analysis of peptoids as antivirals. CVB3 can result in respiratory illness, severe myocarditis, and encephalitis in infected patients.^38^ The clinical relevance of each virus used in this study affirms the need for effective therapies. We observed antiviral activity for seven peptoids with similar characteristics to AMPs against enveloped viruses. Furthermore, we found that PS is a critical lipid target that potentiates membrane disruption by peptoids, allowing the possibility for a selective antiviral mechanism against the broad range of enveloped viruses.

## Results

### Antimicrobial Peptoids Inactivate Enveloped Viruses

**Figure 1:**
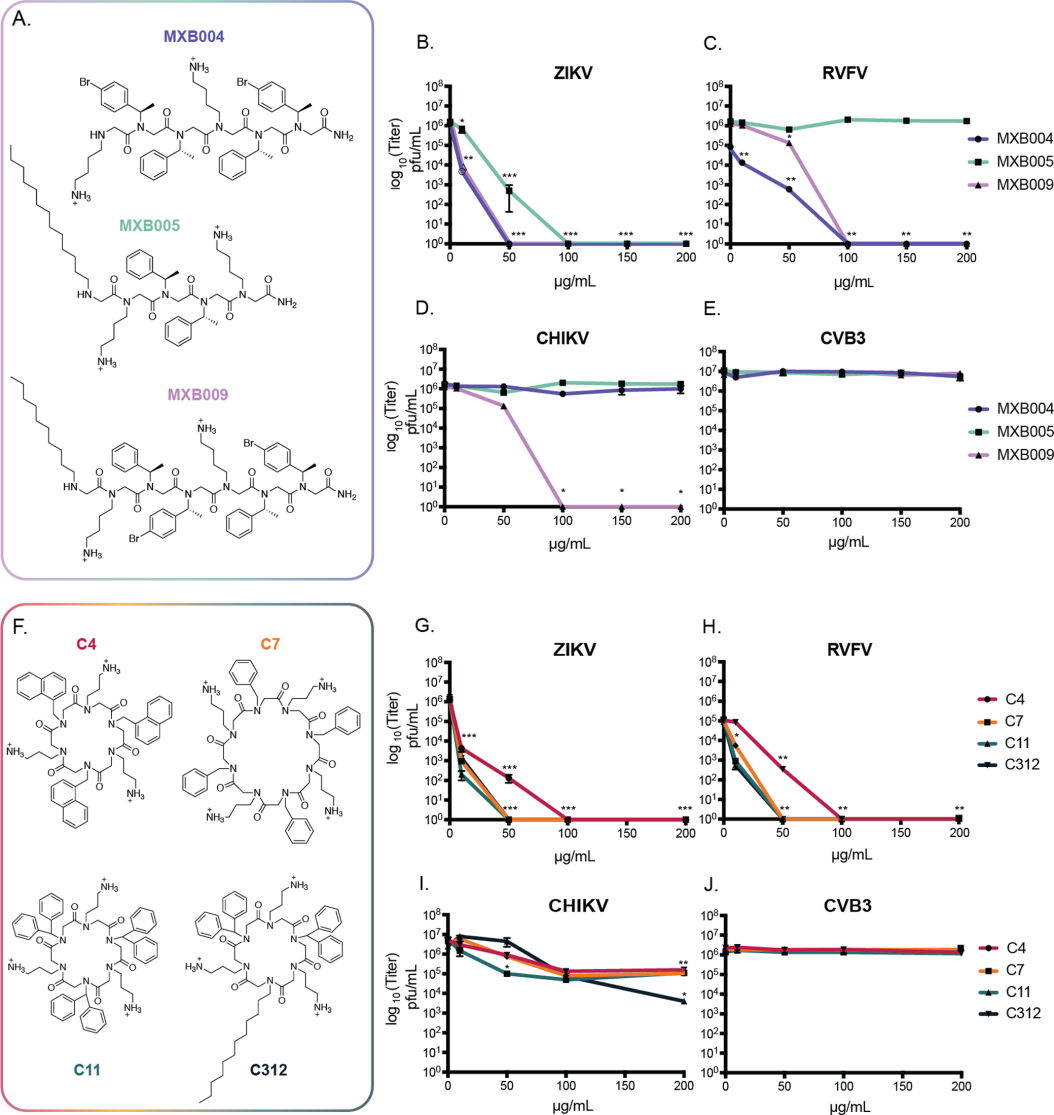
Antimicrobial peptoids inactivate enveloped viruses. The antiviral activities of three linear peptoids (A) were evaluated in vitro against (B) ZIKV, (C) RVFV, (D) CHIKV, or (E) CVB3. Each virus was directly incubated with increasing concentrations of MXB004, MXB005, or MXB009 for 2 h. Virus-peptoid inoculum was collected and viral titers post-peptoid incubation were enumerated via plaque assay. A set of macrocyclic peptoid oligomers (F) were similarly evaluated (ZIKV, panel G; RVFV, H; CHIKV, I; CVB3, J). *p < 0.05, **p < 0.01, and ***p < 0.001 by Student’s *T*-test comparing treatment to untreated conditions (*N* ≥ 3). Error bars represent the standard error of the mean.

We initiated our study by investigating the antiviral activity of linear peptoids MXB004, MXB005, and MXB009 against four different RNA viruses: ZIKV, RVFV MP-12 strain (RVFV MP12), CHIKV, and CVB3. Viruses were pre-incubated directly with peptoids at concentrations of either 10, 50, 100, 150, or 200 μg/mL peptoid. Following a 2 h incubation, residual viral titers were determined in order to measure the antiviral activity (Figure 1). ZIKV was incubated with a range of concentrations of MXB004, MXB005, and MXB009 before measurement of viral titers. ZIKV pre-incubated with 10 μg/mL MXB004 or MXB009 showed a twofold reduction in titers relative to untreated virus (Figure 1B). At concentrations ranging from 50 to 200 μg/mL of MXB004, complete inactivation of ZIKV was observed. MXB009 had similar potency against ZIKV, as no residual titers were measured from 50 to 200 μg/mL peptoid. ZIKV showed little sensitivity to MXB005 at 10 μg/mL; however, at 50 μg/mL a 3-log-fold reduction in viral titers was observed. MXB005 completely inactivated ZIKV at concentrations of 100 μg/mL or greater.

We next explored peptoid-mediated antiviral activity against RVFV. RVFV was pre-incubated with either MXB004, MXB005, or MXB009 for 2 h before measuring viral titers. Both MXB004 and MXB009 exhibited antiviral activity against RVFV (Figure 1C). Sensitivity toward MXB004 and MXB009 was observed at 50 μg/mL peptoid. At concentrations of 100 or 200 μg/mL, no observable titers were measured. MXB005, however, showed little antiviral activity against RVFV. CHIKV was the least sensitive to incubation with peptoids. Viral titers remained unaffected by direct treatment with MXB004 and MXB005; however, MXB009 did exhibit activity (Figure 1D). A log-fold decrease in viral titers was measured after 50 μg/mL of MXB009 incubation. At concentrations of 100 μg/mL MXB009 and higher, no titers were evident. After measuring the antiviral activity against CHIKV, RVFV, and ZIKV, we evaluated the activity against CVB3, a nonenveloped virus. Notably, after direct incubation with MXB004, MXB005, and MXB009, CVB3 titers remained unchanged at all concentrations relative to untreated conditions (Figure 1E).

### Macrocyclic Peptoids Are Antiviral against Enveloped Viruses

In order to further profile the antiviral activity of AMP-like peptoids, we investigated antimicrobial macrocyclic peptoids against ZIKV, RVFV, CHIKV, and CVB3 (Figure 1F). Linear peptoids can be cyclized through head-to-tail amide bond formation. Macrocyclization was shown to enhance the activity of antimicrobial peptoids by constraining their conformations as amphiphiles.^39^ The macrocycles used here incorporate distinct monomer units from the aforementioned linear oligomers described above; however, the physicochemical characteristics between macrocyclic peptoids and linear peptoids are very similar. The side chain groups include a repeating pattern of cationic and hydrophobic residues. The macrocyclic peptoids C4, C7, C11, and C312 were each directly incubated with ZIKV, and viral titers were quantified after 2 h (Figure 1G). At 10 μg/mL, all macrocyclic peptoids substantially reduced viral titers 1000- to 10,000-fold. C7, C11, and C312 completely inactivated ZIKV infectivity at concentrations from 50 up to 200 μg/mL. Compound C4 resulted in total inactivation of ZIKV at slightly higher concentrations of 100 and 200 μg/mL. Cyclic peptoids were then pre-incubated with RVFV, reducing viral titers to undetectable levels (Figure 1H)—C7, C11, and C312 all displayed inhibitory effects at 10 μg/mL peptoid against RVFV, similar to that of ZIKV. At 50 μg/mL and higher, C7, C11, and C312 fully inactivated RVFV. As observed for ZIKV, C4 showed slightly less potent inhibitory affects against RVFV but nonetheless was able to result in successful inactivation of RVFV at a concentration of 100 μg/mL or higher. In contrast, CHIKV showed diminished sensitivity toward macrocyclic peptoids. C312 appeared to have the most dramatic effect against CHIKV with the highest reduction in titers at 200 μg/mL peptoid (Figure 1I). CVB3, a non-enveloped virus, was treated with compounds C4, C7, C11, and C312. Direct incubation of all cyclic molecules with CVB3 resulted in no observable change in viral titers (Figure 1J).

### Peptoid Oligomers Selectively Induce Disruption of Lipid Vesicles in Membranes Incorporating PS

Membrane-active AMPs interact with pathogen lipid surfaces to enhance permeability, resulting in lysis or leakage of essential metabolites or enzymes.^40^ AMP-mediated membrane permeabilization is thought to be a primary mechanism of inactivation against pathogens such as bacteria and enveloped viruses.^41^ Vesicle leakage assays are commonly used to investigate drug-induced membrane disruption.^42^ These assays employ an entrapped fluorophore, which is released upon perturbation and permeabilization of the vesicle membrane. Typically, calcein, a water-soluble fluorescent dye, is encapsulated within the lipid vesicles. Due to calcein self-quenching at high concentrations (>70 mM), changes in fluorescence intensity can be observed when calcein crosses membrane barriers. Calcein, thus, becomes diluted in the surrounding environment so that it is no longer quenched and fluorescence is then observed.^43^ This provides an effective model system for monitoring whether xenobiotic agents can disrupt membrane structures.

**Figure 2:**
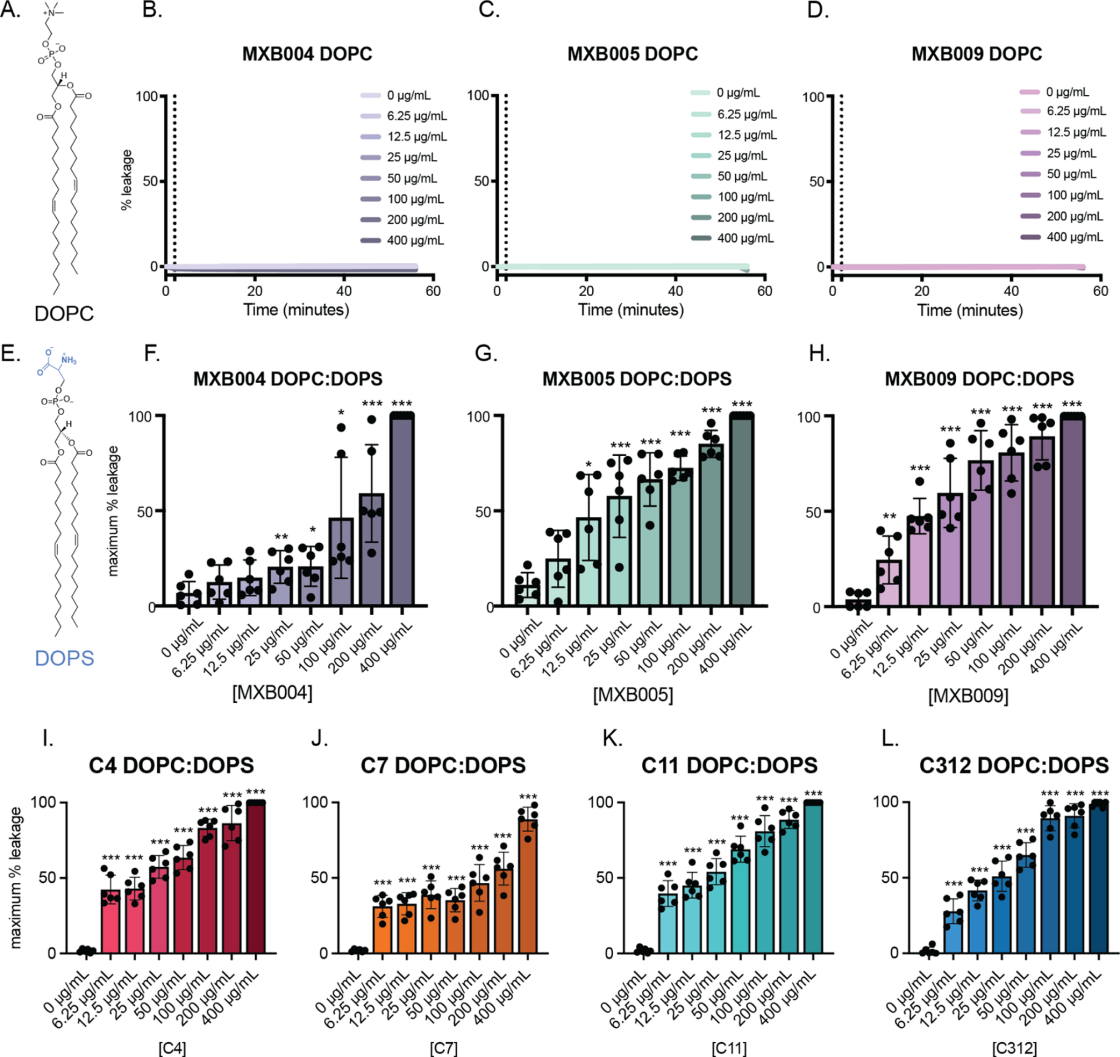
Antimicrobial peptoids induce vesicle leakage in lipid membranes containing PS. Liposome leakage assays utilizing a self-quenching fluorophore were performed to monitor peptoid-mediated membrane disruption. Fluorescence of calcein was measured first in LUVs formed only from DOPC [see the DOPC structure in (A)]. After 4 min, vesicles were treated with (B) MXB004, (C) MXB005, or (D) MXB009 peptoids. Calcein fluorescence was measured for 30 min. At 30 min, 10% Triton was added to achieve maximum fluorescence. Calcein leakage was similarly monitored in phosphatidylcholine/phosphatidylserine [DOPC/DOPS; see the DOPS structure in (E)] formed from LUVs following administration of (F) MXB004, (G) MXB005, (H) MXB009, (I) C4, (J) C7, (K) C11, and (L) C312. Maximum induced leakage in DOPC/DOPS membranes was calculated. Time courses are representative of one experiment. Maximum percent leakage calculations are representative of three preparations of LUVs with technical duplications. *p < 0.05, **p < 0.01, and ***p < 0.001 by Student’s *T*-test comparing treatment with untreated conditions (*N* ≥ 3). Error bars represent standard error of the mean.

To first determine whether peptoids could induce calcein leakage in lipid vesicles, simple large unilamellar vesicles (LUVs) composed solely of phosphatidylcholine (DOPC) lipids were prepared, encapsulating calcein dye at 70 mM. To ensure that these vesicles resembled the sizes of viral particles, dynamic light scattering (DLS) was used to measure the size of prepared LUVs. Measurements by DLS showed that the majority of DOPC vesicles formed were 100 nm in diameter, which is a biologically relevant size regime for modeling many enveloped viruses (Supporting Information Figure 2A).^44,45^ MXB004, MXB005, or MXB009 were added to DOPC LUVs at concentrations ranging from 6.25 to 400 μg/mL peptoid in aqueous buffer (Figure 2). Background fluorescence of calcein-containing LUVs was first measured. After addition of peptoids, membrane fluorescence was monitored for an additional 30 min to measure calcein release. As a positive control, membranes were treated with 10% Triton solution to completely lyse all vesicles and to achieve maximum fluorescence. Percent leakage was calculated by normalizing the minimum and maximum fluorescence values observed in a single experiment for each peptoid.

In the presence of MXB004, MXB005, or MXB009, no enhancement in fluorescence was observed for calcein-encapsulated DOPC LUVs, indicating that no calcein was liberated from these vesicles (Figure 2B–D). These data suggest that MXB peptoids do not generally interact with zwitterionic lipid vesicles to cause subsequent permeabilization. However, bioactivity of AMPs has been suggested to be dependent on membrane lipid composition. This dependence is attributed to variability in lipid–peptide interactions, alteration of membrane curvature, and propensity for pore formation, underscoring the need to evaluate alterations in lipid membrane composition.^46^ To introduce lipid heterogeneity and more closely mimic viral envelope membranes, phosphatidylserine (DOPS) was added to DOPC lipids in a 30:70 molar ratio to form DOPC/DOPS LUVs. As observed by DLS measurements, the DOPC/DOPS LUVs formed also had similar diameters to pure DOPC LUVs (Supporting Information Figure 2B). Increasing concentrations of MXB004, MXB005, and MXB009 were added to DOPC/DOPS LUVs, and calcein leakage was monitored by fluorescence for 30 min. Distinct from DOPC LUVs, calcein leakage in DOPC/DOPS vesicles was observed. MXB004, MXB005, and MXB009 induced spikes in fluorescence immediately following addition to vesicles. Each peptoid induced permeabilization against these anionic vesicles at concentrations as low as 6.25 μg/mL. Peptoids caused complete lysis at the highest concentration of 400 μg/mL. MXB005 and MXB009 appeared to be more robust in destabilizing membrane liposomes, as 50% leakage was observed at concentrations as low as 12.5 μg/mL for these compounds (Figure 2F–H). MXB004 required at least 100 μg/mL to achieve 50% leakage of the DOPS/DOPC LUVs (Figure 2F). These results indicate that bioactivity of peptoid antimicrobials may be specifically exerted against viral membranes containing anionic phospholipids (i.e., those with net negative charge).

In a similar fashion, macrocyclic peptoids (C4, C7, C11, and C312) were tested against liposomes to probe how these compounds engage with lipid constituents in a membrane-mimicking environment. Cyclic peptoids were first tested against vesicles incorporating exclusively PC (Supporting Information Figure 4A–D). At higher concentrations of peptoids, up to 10% leakage was observed for compounds C4, C7, and C11 against DOPC liposomes. Additionally, 15% leakage was measured at concentrations above 200 μg/mL for peptoid C312. Notably, all macrocyclic peptoids induced significantly higher levels of leakage in vesicles containing DOPS relative to DOPC liposomes at concentrations as low as 6.25 μg/mL (Figure 2I–L). All four compounds had very similar profiles of membrane permeabilization against DOPC/DOPS liposomes, with compounds C4 and C11 being the most robust at the lowest concentrations tested. As macrocyclic peptoids were titrated against liposomes, permeabilization of DOPS liposomes substantially increased and maximal fluorophore leakage was observed at the highest peptoid concentrations. While more extensive leakage was observed for macrocycles against PC liposomes compared to treatment with linear peptoids, a clear trend was evident that these antimicrobial peptoids preferentially disrupted vesicles incorporating PS.

### Determination of Critical PS Concentrations for Peptoid-Induced Leakage

The concentration of PS in model membranes may be an important factor when screening peptoids as potent antivirals. Viral lipidomic analysis revealed that HIV type 1 envelope membranes incorporate PS ranging from 9 to 20% of total membrane lipids, dependent upon their progenitor cell.^38^ Phospholipids quantified from three different strains of influenza A virus showed at least 25% of lipids within the envelope to be PS.^46^

**Figure 3:**
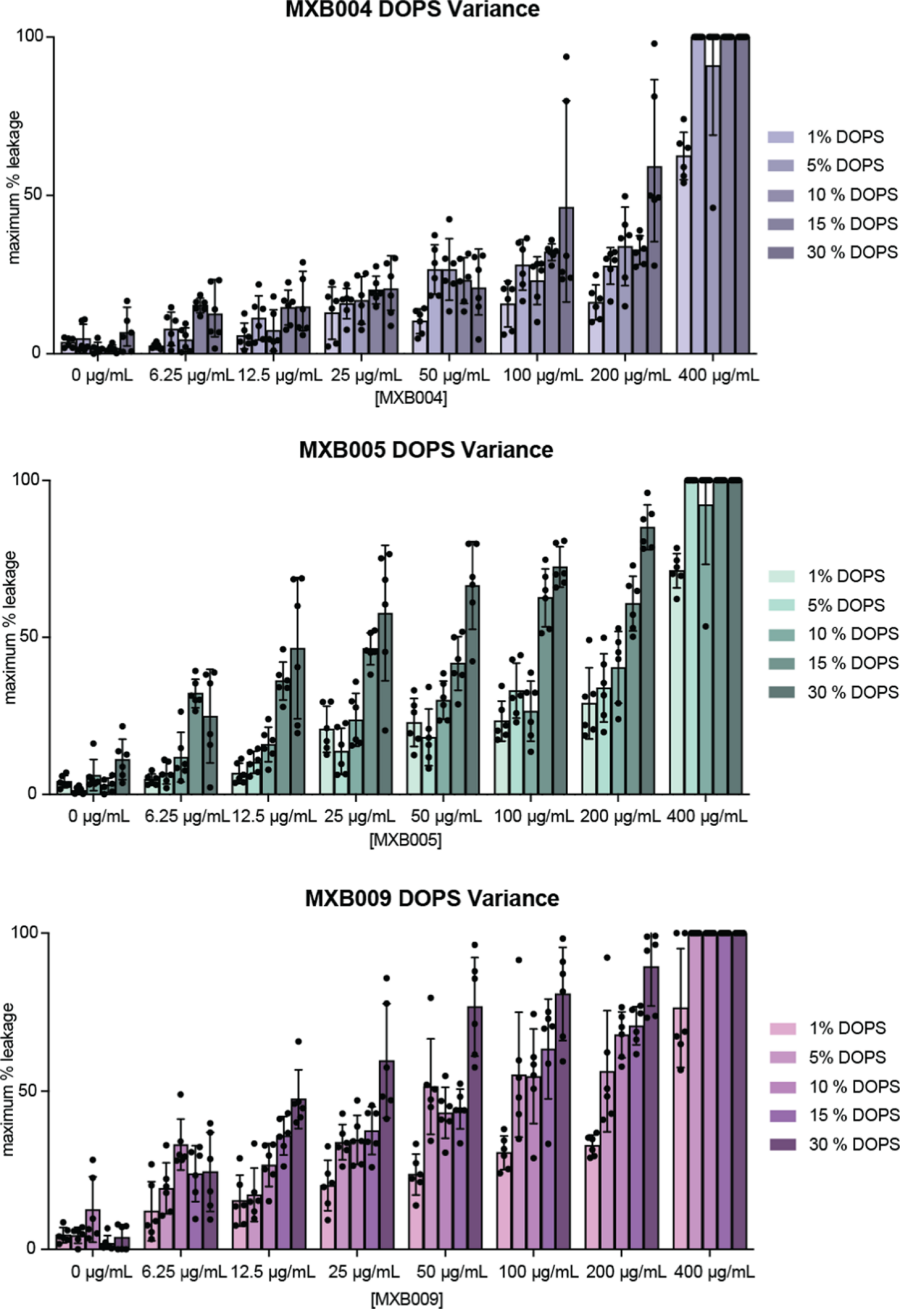
Antimicrobial peptoids can disrupt membranes at low concentrations of PS. To gauge the concentration of PS required for peptoid-mediated membrane disruption, membrane permeabilization was measured by calcein fluorescence leakage in vesicles containing molar ratios of 99:1, 95:5, 85:15, or 70:30 phosphatidylcholine to phosphatidylserine (DOPC/DOPS) lipids. Background calcein fluorescence was first measured for 4 min; then, vesicles were treated with increasing concentrations of (A) MXB004, (B) MXB005, or (C) MXB009 for 30 min. After 30 min, 10% Triton was added to vesicles to induce maximum fluorescence. Maximum percent leakage was calculated by normalizing to minimum and maximum fluorescence values. Leakage experiments are representative of three preparations of LUVs with technical duplicates.

In order to understand how peptoids engage with PS and their specificity toward this lipid, we generated fluorophore-loaded vesicles composed of DOPS/DOPC at 1:99, 5:95, 10:90, 15:85, and 30:70 molar ratios. MXB004, MXB005, and MXB009 were titrated against each of these DOPS-containing vesicles, and calcein leakage was measured. MXB004, MXB005, and MXB009 were all capable of permeabilizing membranes that contained molar ratios of DOPS as low as 1:99 DOPS/DOPC and up to 30:70 DOPS/DOPC; however, some variability was observed (Figure 3). First, MXB004 was the least potent in disrupting liposomes containing variable amounts of DOPS, whereas MXB009 was the most potent. Leakage was observed at all concentrations of MXB004 against liposomes containing only 1 and 5% DOPS, but the leakage profiles for MXB005 and MXB009 were more intense against these liposomes at lower concentrations of peptoid. For both MXB005 and MXB009, modest leakage was observed at all concentrations of peptoid against liposomes containing 1% DOPS; however, more robust leakage was generally observed at concentrations of 5% DOPS or greater (Figure 3B,C). It appears that for both MXB005 and MXB009, 5% of DOPS in the membrane may be the threshold concentration for establishing susceptibility to peptoid-mediated disruption. While the membrane disruption activities varied between each peptoid, these data clearly indicate that the oligomers are able to engage with and disrupt membranes incorporating DOPS at concentrations relevant to those present in many viral envelopes.

### Peptoid Oligomers Induce Leakage in Vesicle Membranes Incorporating Phosphatidylinositol

To elucidate whether MXB004, MXB005, or MXB009 favors interaction with PS or simply requires the presence of an anionic headgroup for membrane permeabilization, lipid vesicles were generated containing phosphatidylinositol (PI). PI is composed of fatty acid tails, a charged phosphate group, and a polar inositol group. The inositol lipids are involved in a variety of signaling pathways but are mainly localized to subcellular membranes and exist in extremely low amounts in the plasma membrane.^47^ By contrast, lipidomic studies indicate that the envelopes of HIV contain PI at concentrations up to 13% of total membrane lipids, depending on the progenitor cell.^48^

DOPC/PI LUVs at a 70:30 molar ratio were monitored by DLS to determine the average vesicle diameter size, which was found to be 110 nm (Supporting Information Figure 2C). MXB004, MXB005, and MXB009 were titrated against DOPC/PI LUVs, and calcein leakage was monitored via fluorescence for 30 min (Supporting Information Figure 3). Vesicle leakage was observed in a concentration-dependent manner for MXB004, MXB005, and MXB009 (Supporting Information Figure 3C). Following the addition of MXB004, MXB005, and MXB009 to DOPC/PI membranes, a sharp increase in fluorescence was observed within the first few minutes, and then a subsequent stabilization of fluorescence was seen. MXB004 established up to 40% calcein release at the maximum concentration of 400 μg/mL (Supporting Information Figure 3E). A concentration dependence as measured by calcein release was shown for MXB004 against DOPC/PI LUVs. The insensitivity observed at higher concentrations of MXB004 in PI-containing vesicles suggests a preferential engagement with PS relative to other anionic lipids. Similar trends of fluorescent leakage were observed for both MXB005 and MXB009 in DOPC/PI membranes. MXB005 resulted in 45% calcein release at 400 μg/mL in PI LUVs, whereas MXB005 was able to induce a similar extent of membrane disruption in DOPS-containing vesicles at only 12.5 μg/mL of peptoid (Supporting Information Figure 3F). A concentration dependence of leakage by MXB005 was seen, similar to that in DOPS vesicles, but the amount of leakage was significantly reduced in PI membranes. Finally, MXB009 was titrated against DOPC/PI LUVs, and the maximum leakage observed was 43% release at 400 μg/mL. MXB009 showed strong permeabilization at a 30-fold lower concentration of peptoid against DOPS-containing LUVs compared to PI-containing vesicles. Overall, calcein release was generally less in LUVs containing PI relative to PS-containing LUVs, indicating that antiviral peptoids may have a greater selectivity toward PS over PI.

DOPC/PI liposomes were subsequently tested for sensitivity to macrocyclic peptoids C4, C7, C11, and C312 in order to monitor the scope of lipid engagement by measuring membrane permeability (Supporting Information Figure 4). Following the addition of all macrocycles, sharp increases in fluorescence were observed even at the lowest concentrations of peptoid. C4 induced approximately 45% leakage at 6.25 μg/mL, and 80% leakage was observed at the highest concentration of 400 μg/mL (Supporting Information Figure 4E). Compounds C7 and C11 followed similar trends and were capable of inducing over 75% leakage in these liposomes (Supporting Information Figure 4F–G). Interestingly, C312 induced maximum leakage in PI-containing vesicles at the higher range of peptoid added (Supporting Information Figure 4H). A slight preference for PS over PI was observed for peptoid macrocycles permeabilizing liposomes; however, the leakage profiles were substantially more robust in PI vesicles incubated with macrocycles relative to linear peptoid. The promiscuous activity observed with macrocyclic peptoid-mediated membrane permeabilization may account for the greater antiviral potency against enveloped viruses.

### Peptoid Treatment Alters Viral Membrane Integrity

To further elucidate if antiviral peptoids perturb viral membranes, we investigated the stability of two enveloped viruses through the measurement of viral genome levels and viral envelope protein levels. Proteins present within the viral membrane are susceptible to degradation after treatment with membrane disrupting agents. Viral genomes, however, are protected by a viral protein capsid, which may remain intact following membrane disruption. If the integrity of the viral protein capsid is also compromised after peptoid treatment, the viral RNA will quickly be degraded, and quantification of the viral genome will be reduced significantly.

**Figure 4:**
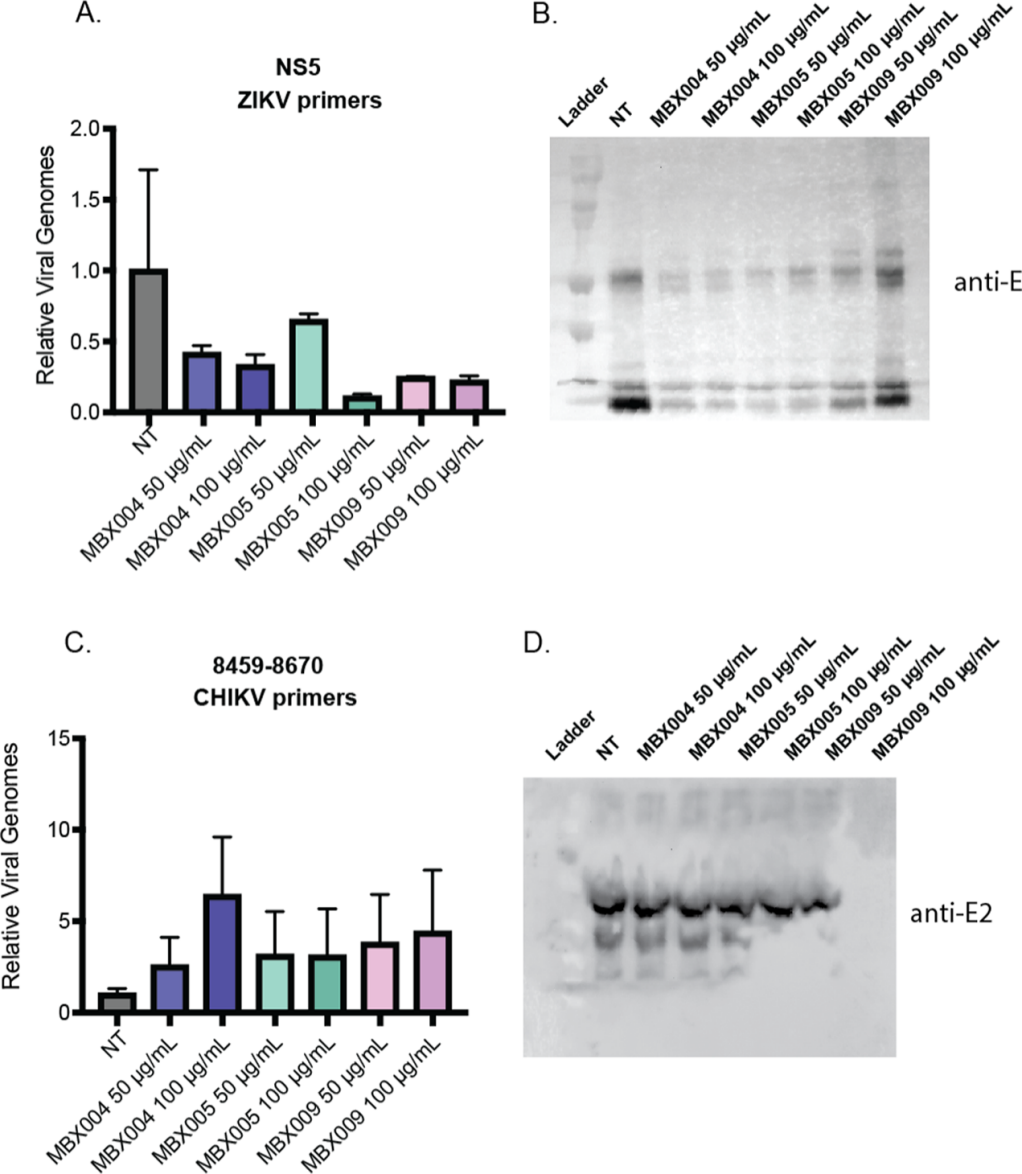
Viral envelopes degrade with peptoid treatment. To measure if antiviral peptoids have the capability to disrupt viral membranes, both viral genomes and viral envelope protein were quantified after peptoid incubation. ZIKV was directly incubated with either 50 or 100 μg/mL of MXB004, MXB005, or MXB009 for 2 h and (A) viral RNA levels were quantified via RT-qPCR after peptoid incubation. Additionally, (B) ZIKV E protein was visualized via Western blot after incubation with or without peptoid. CHIKV was directly incubated with 50 or 100 μg/mL and (C) RNA levels were quantified via RT-qPCR after peptoid incubation. (D) CHIKV E protein was visualized via Western blot before and after incubation with MXB004, MXB005, or MXB009. Error bars represent standard error of the mean.

Peptoids were directly incubated with virus for 2 h, and following incubation, the amount of viral RNA was quantified via RT-qPCR. Additionally, viral envelope protein levels were measured via Western blot after peptoid–virus incubation to monitor membrane stability. We directly incubated ZIKV viral stocks with 50 or 100 μg/mL of MXB004, MXB005, or MXB009. After incubation with peptoids, ZIKV RNA was quantified via RT-qPCR to measure viral genomes at two separate regions (Figure 4A). At all concentrations of MXB peptoids, viral genome levels remained relatively unchanged compared to untreated viruses at both the RNA genome segments, suggesting that viral genomes remained intact despite significant reduction in viral titer.

Interestingly, when ZIKV envelope protein E was analyzed via Western blot, degradation of protein was observed at both concentrations of MXB004, MXB005, and MXB009 (Figure 4B). These data suggest that peptoid-mediated envelope disruption destabilizes viral envelope proteins, while the viral genomes remain intact within the capsid, consistent with a role for peptoids in disrupting the viral envelope. We confirmed these results by measuring CHIKV membrane integrity after incubation with antiviral peptoids. MXB004, MXB005, and MXB009 were directly incubated with CHIKV viral stocks at either 50 or 100 μg/mL peptoid. CHIKV viral RNA was extracted and quantified via RT-qPCR after peptoid incubation. CHIKV viral genome levels remained relatively unchanged at both concentrations of MXB004, MXB005, or MXB009 (Figure 4C). To gauge whether peptoid interaction with virus was altering the integrity of the membrane, we investigated the stability of CHIKV envelope protein E2. At both concentrations of MXB004 and MXB005, E2 protein levels remained unchanged relative to untreated CHIKV E2. Degradation of E2 was observed solely with pre-treatment of MXB009 (Figure 4D). We correlate this observation to the fact that only MXB009 reduced viral titers of CHIKV, underscoring a higher potency of MXB009 for enveloped viruses than other peptoids (Figure 4C). Similar to experiments conducted on ZIKV, it appears that MXB009 can alter membrane stability during the incubation, yet the protein capsid surrounding the genome may remain intact as genome levels are not significantly changed. Overall, it appears that peptoids directly act on envelopes to disturb viral membranes and neutralize virus.

### Viral Envelopes Engineered to Augment PS Content Are More Susceptible to Antiviral Peptoids

**Figure 5:**
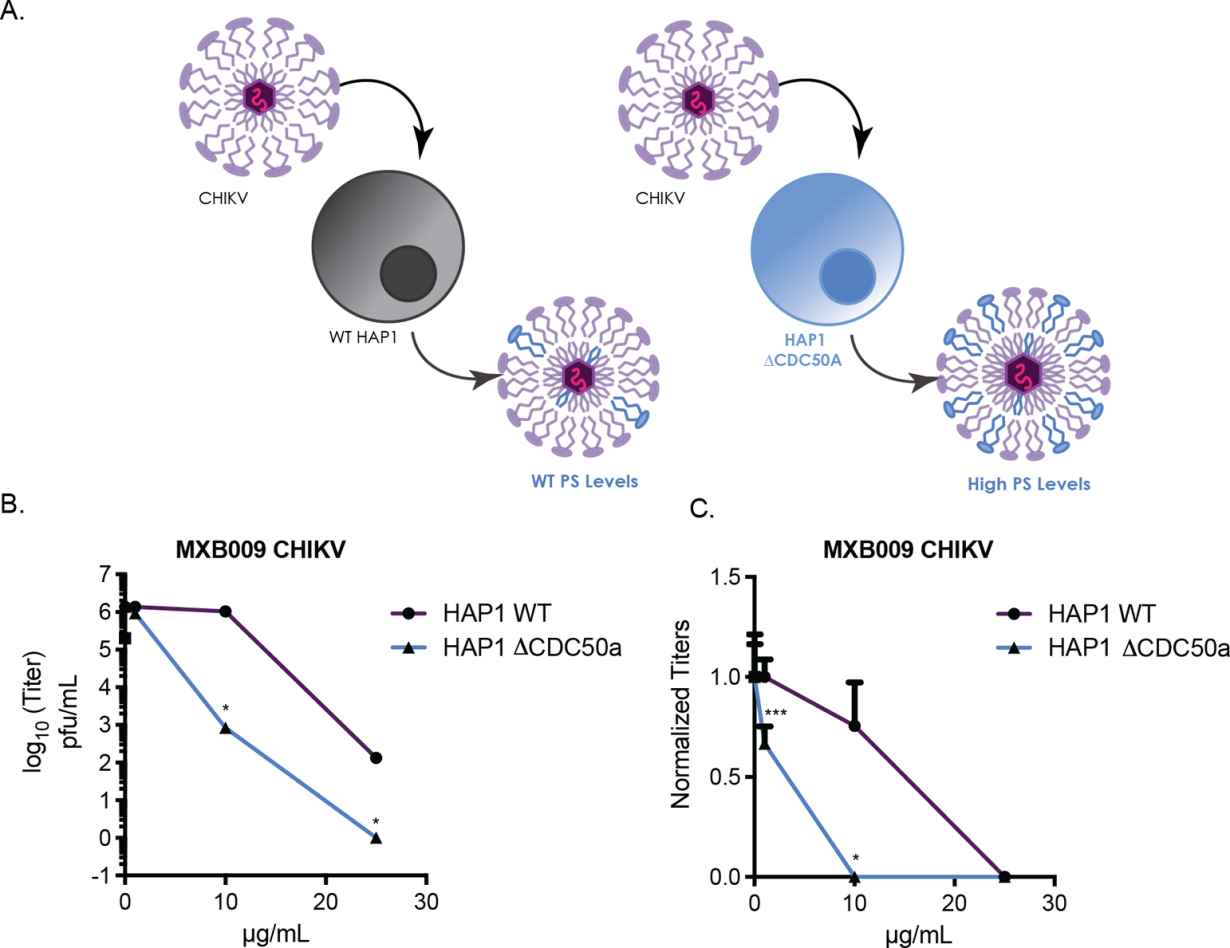
CHIKV PS levels influence antimicrobial peptoid sensitivity. (A) A knockout cell system generating viruses with elevated amounts of viral membrane PS was used to measure the importance of PS in the antiviral mechanism of amphiphilic peptoids. Increasing concentrations of (B) MXB009 were directly treated with CHIKV generated from HAP1 WT cells or HAP1 ΔCDC50a cells for 2 h. Viral supernatant was collected, and viral titers were quantified via plaque assay. (C) Viral titers were normalized to minimum and maximum titers. *p < 0.05, **p < 0.01, and ***p < 0.001 by Student’s *T*-test comparing treatment with untreated conditions (*N* ≥ 3). Error bars represent standard error of the mean.

To probe whether the antiviral activity of MXB compounds was dependent on the presence of PS, we generated two preparations of CHIKV virions with varying concentrations of PS. CHIKV was propagated from either WT cells or CDC50a knockout HAP1 cells. CDC50a is an ATP-flippase that translocates PS from the outer to the inner leaflet of the plasma membrane. CDC50a knockout cells have higher concentrations of PS on the outer cell membrane relative to wild-type cells. Viruses generated from ΔCDC50a cells have been shown to generate higher concentrations of external PS on viral membranes relative to viral stocks generated from WT cells.^49^ To measure whether PS levels would influence the antiviral activity of MXB peptoids, three concentrations of MXB009 were directly incubated with CHIKV stocks generated from either WT HAP1 cells or ΔCDC50a cells and viral titers were quantified after peptoid incubation (Figure 5A). Interestingly, at 10 μg/mL MXB009, CHIKV generated from ΔCDC50a cells showed higher sensitivity toward peptoid incubation relative to CHIKV virus propagated from WT cells (Figure 5B). MXB009 resulted in a 3-log fold reduction of CHIKV viral titers from ΔCDC50a cells, whereas titers of WT CHIKV appeared unchanged at 10 μg/mL MXB009. At 25 μg/mL, MXB009 was able to completely inhibit CHIKV infection in virus generated from ΔCDC50a cells with more than a 6-log reduction in titers relative to untreated virus. However, WT CHIKV incubated with MXB009 at 25 μg/mL was reduced to 2000 pfu/mL, 2-log higher than CHIKV from ΔCDC50a cells at the same concentration. These results indicate that MXB009 can more potently inhibit CHIKV when concentrations of PS are increased within the viral membrane. In conjunction with vesicle leakage assays, the results confirm that the presence of negatively charged lipid PS, in particular, confers susceptibility to membrane disruption and inhibition of viral infectivity by antimicrobial peptoids.

## Discussion

The evaluation of host defense peptides as antiviral agents has recently gained momentum, yet the clinical implementation of these peptides as therapeutic agents is limited. A major barrier to the use of AMPs is their toxicity toward host cells.^50^ While AMP agents show potent activity against pathogens, hemolytic assays often reveal disruption of eukaryotic cells as well.^51^ Furthermore, AMPs are unstable in the host environment due to their susceptibility to proteolytic degradation.^52^ High concentrations and consistent dosing are required for effective use of AMPs; however, this approach creates unwanted toxicity.^53,54^ Strategies to incorporate unnatural amino acids or stereoisomers have been used to overcome these shortcomings, but these tactics are only moderately effective.^55^ Beyond the properties of AMPs themselves, the targets of antiviral peptides are of concern. Most natural and synthetically derived host defense antiviral peptides either non-specifically target membrane structures or target specific viral proteins.^7,56^ The latter presents concerns of therapeutic resistance as viral protein targets can evolve rapidly. Non-specific targeting of the viral membrane can lead to host-toxicity. Altogether, there is a critical need to identify specific, non-toxic antiviral agents that directly engage conserved viral components.

Our results indicate that certain amphiphilic peptoid oligomers can mimic AMPs and exert antiviral activity through a membrane disruptive mechanism. Furthermore, the presence of PS appears to be critical for peptoid-mediated activity. The association of activity with specific lipid constituents provides an opportunity for selectivity against pathogen membranes. We investigated the antiviral activity of three linear amphiphilic peptoids, MXB004, MXB005, and MXB009, against four viruses: ZIKV, RVFV, CHIKV, and CVB3. Direct incubation of these viruses with the peptoid compounds reduced infectious virus titers to variable degrees. Some of this variability could be due to the differences in the physiochemical properties of the individual compounds. Most notably, all three enveloped viruses were susceptible to peptoid-mediated inactivation, whereas CVB3, the only non-enveloped virus, remained impervious to peptoid treatment. Previous studies performed by Diamond et al. show that MXB004, MXB005, and MXB009 are potent antiviral agents against two other enveloped viruses: herpes simplex virus type-1 (HSV-1) and SARS-CoV-2.^20^ The Diamond et al. study additionally showed by cryo-EM that viral membranes were extensively disrupted following peptoid incubation; however, little to no in vitro cytotoxicity of oral epithelial cells treated with peptoids up to concentrations of 256 μg/mL was observed.^20^ Taken together, these results begin to meet a critical need for antiviral therapies: the selective targeting of conserved viral factors.

Previously studied cyclic antimicrobial peptoids (C4, C7, C11, and C312) were also tested against enveloped viruses to determine their antiviral activity.^57^ Both ZIKV and RVFV showed high sensitivity toward all four cyclic compounds. C4, C7, C11, and C312 were also capable of modestly reducing CHIKV titers; however, complete inactivation via these peptoids was not observed. The fact that we observed activity against all enveloped viruses prompted us to evaluate the molecular mechanisms associated with engagement and disruption of membrane structures.

In vitro calcein release assays revealed that peptoids can disrupt lipid membranes similar to those found on enveloped viruses. When titrated against PC-vesicles, linear peptoids were unable to induce membrane disruption and subsequent fluorophore leakage; however, when PS was introduced into vesicles, robust leakage was observed. Macrocyclic compounds displayed a similar trend in selectivity for membranes with PS; however, slightly greater leakage was observed for vesicles containing exclusively DOPC compared to linear peptoids. Notably, PS is asymmetrically distributed on the inner leaflet of eukaryotic cell membranes.^58^ In resting cells, ATP-dependent flippases rigorously segregate PS toward the cytosol.^59^ In contrast, enveloped viruses lack enzymes that control the dynamics of lipids in membranes. After budding viruses acquire their lipids from the host to form viral membranes, PS becomes extensively distributed between inner and outer membrane leaflets.^60^ The difference in the distribution of PS between virus and host presents an avenue for selectivity.

The interaction between lipid vesicles and antiviral peptoids may be initiated by the electrostatic interactions between the anionic phospholipid head group and the cationic side chains found on antiviral peptoids. Previous studies have demonstrated that some antibacterial peptoids can disrupt membranes containing palmitoyl-2-oleoyl-*sn*-glycero-3-phospho-(1′-*rac*-glycerol) (POPG), an anionic lipid found in high concentrations in bacterial membranes.^61^ A favorable driving force for membrane permeabilization may initially be electrostatic; however, multiple modes of action for AMPs have been described. Predicted modes of action such as the barrel-stave model or the toroidal pore model suggest that electrostatics alone are insufficient for destabilizing pathogen lipid bilayers.^62^ The full range of mechanisms of action for AMPs is still widely debated, and further work on both peptides and peptoids that engage pathogen membranes is needed.

Peptoid engagement with viral membranes may also include interaction with other lipids, as amphiphilic peptoids were capable of modestly disrupting vesicles incorporating PI. Thus, differences in viral lipidome may explain why peptoid-mediated antiviral activity varied between ZIKV, RVFV, and CHIKV. For example, MXB004 and MXB009 were able to similarly inhibit ZIKV at the same concentrations of peptoid; however, CHIKV was only susceptible to MXB009 incubation. Evidence from studies of *Flaviviruses* and *Alphaviruses* along with other viruses such as HIV and influenza suggest that many enveloped viruses establish lipid compositions that are distinct from the membrane of the host.^23,25^ Differences in lipid constituents within the envelopes of ZIKV, CHIKV, and RVFV may be one explanation for the variability of inhibition during peptoid treatment. The extent of accessibility of membrane lipids on the virion surface may also play a role in determining the activity of this family of antiviral compounds. ZIKV, RVFV, and CHIKV virions are extensively coated in class II glycoproteins, whereas previously studied viruses, SARS-CoV-2 and HSV-1, express class I glycoproteins at membrane surfaces.^63−66^ Differences in the glycoprotein content could alter accessibility to the viral envelope.

We probed the role of PS and its influence on viral membrane susceptibility to peptoids by altering the concentration of PS in CHIKV envelopes. This was accomplished by propagating CHIKV in a CDC50a knockout cell system. CDC50a, an ATP-dependent flippase, translocates PS to the cytosolic leaflet of the plasma membrane. Knockout of CDC50a results in unrestricted diffusion of PS to the outer leaflet of the plasma membrane. Viruses propagated in this cell line incorporate increased levels of PS relative to viruses harvested from WT cells. We found increased sensitivity to the peptoid compounds in virions incorporating elevated levels of PS in their membranes. We conclude that antimicrobial peptoids act in a selective manner against viral envelopes by preferentially targeting PS on the outer membrane, establishing a direct mechanism of action against a conserved viral target.

We observed differences in antiviral activity for individual peptoids against ZIKV, RVFV, and CHIKV. A comparison between peptoids revealed varying degrees of potency. The compound MXB009 inactivated all three viruses across a range of concentrations. In contrast, MXB005 was selectively active against ZIKV. MXB004 showed activity against ZIKV and RVFV, but not against CHIKV. The differences in antiviral peptoid effectiveness may be ascribed to variations in the monomer sequences for MXB004, MXB005, and MXB009. Macrocyclic peptoids also exerted antiviral activity against enveloped viruses. The physicochemical features of the peptoids used in this work have been extensively studied to optimize antibacterial and antifungal activity while maintaining low cytotoxicity against eukaryotic cells, and only recently have these peptoids been evaluated against viruses. Nonetheless, the data presented suggests a promising design strategy for antiviral peptoids: specific targeting of PS.

In this study, we investigated the antiviral profiles of seven amphiphilic peptoid sequences against three enveloped viruses and one non-enveloped viruses. Selectivity for viruses containing a lipid envelope was observed for all seven antiviral peptoids. Membrane disruption experiments revealed that linear and cyclic amphipathic sequences engaged with vesicles that contain anionic phospholipids, including a high specificity for PS-containing vesicles. Virions containing elevated amounts of PS were more sensitive to peptoid-mediated inactivation, suggesting a critical role of PS in the antiviral mechanism of these compounds. As viruses obtain lipids from their host during replication and do not genetically encode their own lipid constituents, targeting the membrane bilayer of enveloped viruses offers a pathway toward effective therapeutics, which may prevent the generation of resistant variants. Additionally, these compounds act directly on virus particles to disrupt their membranes, establishing potential countermeasures against newly emerging viral threats.

## Materials and Methods

### Materials

Dichloromethane (DCM), acetonitrile (ACN), and HPLC grade water were supplied by Pharmco. 2-Chlorotrityl chloride resin, dimethylformamide (DMF), bromoacetic acid, *N*,*N*-diisopropylethylamine (DIEA), diisopropylcarbodiimide (DIC), trifluoroacetic acid (TFA), triisopropylsilane (TIPS), PyBOP, benzylamine, 1-napthylmethylamine, and benzhydrylamine were provided by Millipore Sigma. *N*-Boc-1,3-diaminopropane was provided by Matrix Scientific.

### Peptoid Oligomers

MXB004, MXB005, and MXB009 peptoid oligomers were generously provided by the Barron Lab at Stanford University. Peptoids were stored at stock concentrations of 1 mg/mL in 10 mM Tris–HCl, 50 mM NaCl (pH 7.4) buffer, and the concentrations were determined by dry weight. Macrocyclic peptoids were synthesized through sub-monomer solid-phase synthesis. 2-Chlorotriyl chloride resin was first swelled and washed in DCM. The first coupling step included 5 equiv of bromoacetic acid with 10 equiv of DIEA on a shaker platform for 20 min at RT. After the initial bromoacetylation, resin was washed extensively with DCM followed by washing with DMF. A solution of 20 equiv of desired amine in DMF was added to resin to introduce the first chain of the peptoid oligomer for 30 min to 1 h. The subsequent bromoacetylation steps were performed with 10 equiv of bromoacetic acid and 10 equiv of DIC. An iterative process of amine displacement and bromoacetylation was repeated until the desired oligomer length was achieved. The resin-bound peptoid was cleaved in a solution of 10% acetic acid in DCM at room temperature for 1 h. The cleavage solution was dried under N_2_ gas, dissolved in a mixture of 50% ACN/H_2_O, frozen, and lyophilized to yield white powders. Crude linear peptoids were then used for subsequent cyclization. Peptoids were first dissolved in dry DMF followed by the addition of 10 equiv DIEA and subsequently with 5 equiv of PyBOP and stirred overnight at RT. DMF was removed by rotary evaporation. Crude cyclized peptoids were dissolved in a cleavage cocktail of 95% TFA, 2.5% TIPS, and 2.5% H_2_O to remove *tert*-butyl protecting groups on the lysine-like side chains. Crude cyclic peptoids were stirred for 2 h, and the solution was dried under nitrogen gas. The oily product was then dissolved in 50% ACN/H_2_O and purified with reverse-phase HPLC using water and ACN as mobile phases. HPLC fractions were evaluated for purity by HPLC analysis using a reversed-phase analytical column (Eclipse Plus C18, 3.5 μm, 4.6 × 100 mm) with a linear gradient of ACN (0.1% TFA) into H_2_O (0.1% TFA) over 20 min at a flow rate of 0.7 mL/min, using the Agilent HPLC system (model: 1260 Infinity) with a UV–vis wavelength detector set at 220 nm. All compounds used were assessed with >95% purity. Molecular weight of each product was confirmed by using an Agilent 6120 single quadrupole LC–MS spectrometer.

### Preparation of LUVs

LUVs were prepared using 1,2-dioleoyl-*sn*-gylcero-3-phosphocholine (DOPC; Avanti Polar Lipids) alone or in combination with either 1,2-dioleoyl-*sn*-glycero-3-phospho-l-serine (DOPS; Avanti Polar Lipids) or 1-palmitoyl-2-oleoyl-*sn*-glycero-3-phosphoinositol (PI; Avanti Polar Lipids) in a 70:30 molar ratio, as previously described. Stocks of DOPC, DOPS, or PI were mixed to reach final concentrations of 10 mM total lipid. DOPC, DOPC/DOPS, or DOPC/PI solutions were evaporated under dry N_2_ and dried in a desiccator. Lipid films were resuspended in 10 mM Tris–HCl, 50 mM NaCl buffer (pH 7.4), supplemented with or without 70 mM calcein (Millipore Sigma). To reduce vesicle size, LUVs were subject to five freeze–thaw cycles from −80 to 40 °C. LUVs were bath sonicated for 30 min or until the solutions were entirely cleared. LUVs were filtered 20 times through a 0.2 μm pore filter (Anatop 10, Whatman). Vesicles hydrated in calcein-containing buffer were purified twice through PD-10 Desalting Columns (GE Healthcare) containing Sephadex G-25 resin to liberate excess calcein. LUVs were eluted from columns with 10 mM Tris–HCl, 50 mM NaCl buffer (pH 7.4). Final concentrations of vesicles were calculated using a colorimetric phospholipid quantification kit as described by the manufacturer (Millipore Sigma). LUVs were stored at 4 °C up to one week.

### Size Determination of LUVs by DLS

DOPC, DOPC/DOPS, or DOPC/PI vesicle diameters were measured via DLS using a Malvern Zetasizer Nano (New York University; Shared Instruments Facilities). Samples were prepared in polystyrene cuvettes after filtration to final concentrations of 2.5 mM LUVs in 10 mM Tris–HCl, 50 mM NaCl (pH 7.4) buffer. Samples were recorded using the size measurement SOP for 20 counts for two separate runs. Size intensity distributions were averaged after two runs.

### Calcein Leakage Assays

Calcein release assays were performed in 96-well clear bottom microtiter plates and were monitored by a plate reader (Molecular Devices ID5), as previously described.^67^ Calcein-encapsulated LUVs were added at concentrations of 20 μM for DOPC-alone vesicles, 12.5 μM DOPC and 5.5 μM DOPS for DOPC/DOPS LUVs, and 12.5 μM DOPC and 5.5 μM PI for DOPC/PI LUVs. Varying concentrations of three peptoid molecules, MXB004, MXB005, and MXB009, in 10 mM Tris–HCl, 50 mM NaCl (pH 7.4) buffer were added in duplicate to calcein-encapsulated LUVs. Calcein release was compared to untreated vesicles diluted in 200 μL of buffer. Calcein fluorescence was measured every 3 min for 1 h at 37 °C at an excitation of 485 nm and an emission of 530 nm. To determine maximum leakage, 10% Triton was added to result in vesicle lysis and completely freed calcein. Membrane permeabilization as a function of calcein leakage was calculated using the following equation
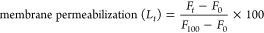
1*F*_*t*_ is the measured fluorescence at time *t*, *F*_0_ is the fluorescence measured at time zero, and *F*_100_ is the maximum fluorescence.

### Cell Culture

Cells were maintained in Dulbecco’s modified Eagle’s medium (DMEM; Life Technologies) with bovine serum and penicillin–streptomycin at 37 °C in 5% CO_2_. Vero cells (BEI Resources) were supplemented with 10% new-born calf serum (NBCS; Thermo-Fischer). We were kindly gifted human near-haploid cells (WT-HAP1 and HAP1 ΔCDC50a) from Dr. Melinda A. Brindley.^68^ VeroS and VeroS KO lines were maintained with DMEM supplemented with 10% new-born calf serum. HAP1 and HAP1 KO lines were cultured in Iscove’s modified Dulbecco’s medium supplemented with 10% fetal bovine serum.

### Incubation and Enumeration of Viral Titers

RVFV MP-12 strain was derived from the first passage of virus in Huh7 cells.^69^ ZIKV (MR766) and CHIKV (NR-13222) and human rhino virus 1A were provided by Dr. Bill Jackson and were derived from the first passage of virus in Vero cells. ZIKV (African strain) and CHIKV (BSL2 vaccine candidate strain) were obtained from Biodefense and Emerging Infections (BEI) Research Resources. CVB3 (Nancy strain) was derived from the first passage of virus in HeLa cells. Viral stocks were maintained at −80 °C. For peptoid incubation experiments, virus was diluted to desired concentration in serum-free DMEM. Viruses were incubated with varying concentrations of peptoids for 2 h at 37 °C, or longer where indicated. Supernatants were collected from RVFV, ZIKV, CHIKV, and CVB3. To quantify viral titers, dilutions of viral supernatant were prepared in serum-free DMEM and used to inoculate a confluent monolayer of Vero cells for 10–15 min at 37 °C. Cells were overlaid with 0.8% agarose in DMEM containing 2% NBCS. CVB3 samples were incubated for 2 days, CHIKV samples were incubated for 2 days, RVFV samples were incubated for 4 days, and ZIKV samples were incubated for 4 days at 37 °C. Following specified incubation, cells were fixed with 4% formalin and plaques were revealed with crystal violent solution (10% crystal violet; Sigma-Aldrich). Plaques were enumerated and used to back-calculate the number of plaque-forming units per milliliter of collected volume.

### RNA Purification, cDNA Synthesis, and Viral Genome Quantification

Viral supernatant after peptoid incubation was collected, and Trizol reagent (Zymo Research) was directly added. Lysate was collected, and RNA was purified according to the manufacturer’s protocol utilizing the Direct-zol RNA Miniprep Plus Kit (Zymo Research). Purified RNA was used for cDNA synthesis using the High-Capacity cDNA Reverse Transcription Kit (Thermo Fischer), according to the manufacturer’s protocol. Viral genomes were quantified as previously described.^70^ Primers were designed against various regions of the ZIKV and CHIKV genomes, and primer sequences (IDT) are shown in Table 1. Values were normalized to untreated conditions relative viral genomes ratio.

**Table 1:** RT-qPCR Primer Sequences

ZIKV NS5 forward	5′-AAATACACATACCAAAACAAAGTGGT-3′
ZIKV NS5 reverse	5′TCCACTCCCTCTCTGGTCTTG-3′
ZIKV 5865 forward	5′-CCCTCAAGTATAGCAGCAAGAG-3′
ZIKV 5985 reverse	5′-TGAGTTGGAGTCCGGAAATG-3′
CHIKV 8459 forward	5′-TGCTTGAGGACAACGTCATGAG-3′
CHIKV 8670 reverse	5′-GTCTGTCGCTTCATTTCTGATG-3′
CHIKV 9648 forward	5′-AGTTGTGTCAGTGGCCTCGTTC-3′
CHIKV 9861 reverse	5′-AAAGGTTGCTGCTCGTTCCAC-3′

### Western Blot

ZIKV and CHIKV were incubated with varying concentrations of peptoids, and samples were collected with Bolt LDS Buffer and Bolt Reducing Agent (Invitrogen) and run on polyacrylamide gels. The gels were transferred to membranes using the iBlot 2 Gel Transfer Device (Invitrogen). Membranes were probed with primary antibodies for CHIKV envelope protein E2 (1:1000, BEI Resources) and ZIKV envelope protein E (1:1000, EastCoast Bio). Membranes were treated with SuperSignal West Pico PLUS Chemiluminescent Substrate (Thermo Fisher Scientific) and visualized on a ProteinSimple FluorChem E Imager.

### Statistical Analysis

Prism 9 (GraphPad) was used to generate graphs and preform statistical analysis. One-tailed Student’s *t*-test was used with *a* = 0.05. *P*-values were derived from *Z* scores with two-tails and *a* = 0.05. Statistical details are in individual figure legends with NS *p* > 0.05, *p < 0.05, **p < 0.01, and ***p < 0.001.

## Supporting Information Available

Mass spectrometry and analytical HPLC traces of synthesized macrocyclic peptoids, DLS measurements of LUVs, calcein leakage experiments of PI LUVs, and calcein leakage experiments of PC and PI LUVs with macrocyclic peptoids (PDF)

## Abbreviations

AMP: antimicrobial peptide
ZIKV: Zika virus
RVFV: Rift Valley Fever virus
CHIKV: chikungunya virus
CVB3: coxsackie B3 virus
LUV: large unilamellar vesicle
PC: phosphatidylcholine
PS: phosphatidylserine
PI: phosphatidylinositol
