# Supplementary material for: Peptidomimetic Oligomers Targeting Membrane Phosphatidylserine Exhibit Broad Antiviral Activity
Source: ACS Infect Dis. 2023 Aug 2;9(8):1508–22. doi: 10.1021/acsinfecdis.3c00063 (PMC10425984; doi:10.1021/acsinfecdis.3c00063)
Supplement: Supplementary file 1 — id3c00063_si_001.pdf [file id3c00063_si_001.pdf]

Supporting Information

Peptidomimetic oligomers targeting membrane phosphatidylserine exhibit broad antiviral activity

Authors: Patrick M. Tate[1], Vincent Mastrodomenico[2], Christina Cunha[2], Joshua McClure[3], Annelise E. Barron[3,4], Gill Diamond[5], Bryan C. Mounce[2], Kent Kirshenbaum[1,3]*

1. Department of Chemistry, New York University, New York, NY 10003, USA

2. Department of Microbiology and Immunology, Loyola University Chicago Medical Center, Maywood, IL 60130, USA

3. Maxwell Biosciences, Austin, TX 78738, USA

4. Department of Bioengineering, Stanford University, Stanford, CA 94305, USA

5. Department of Oral Immunology and Infectious Diseases, University of Louisville School of Dentistry, Louisville, KY 40292, USA

*Corresponding Author: kent@nyu.edu

This document contains five pages and four figures.

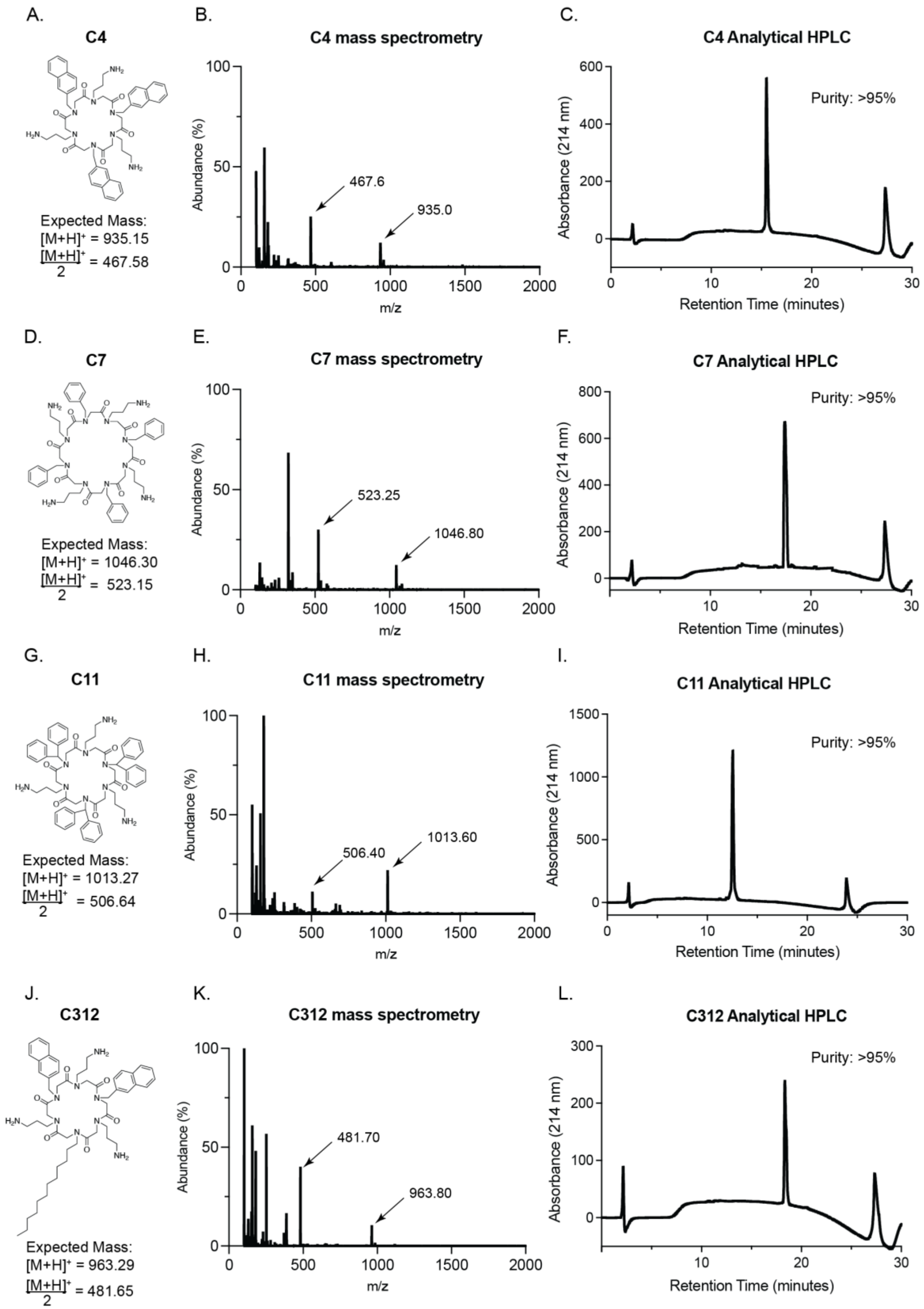

Figure S1. Mass spectrometry and analytical HPLC traces of macrocyclic peptoids. Structure of (A) C4 (D) C7 (G) C11 and (J) C312 with expected masses for mass spectrometry. Mass spectrometry spectra were recorded to confirm compound size for (B) C4 (E) C7 (H) C11 and (K) C312. Analytical HPLC was performed to determine purity of (C) C4 (F) C7 (I) C11 and (L) C312.

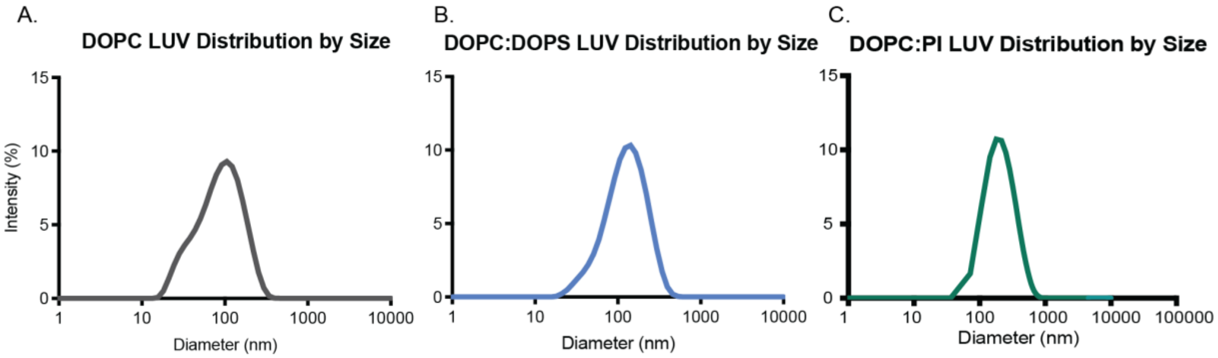

Figure S2. Size determination of Large Unilamellar Vesicles. To determine the diameter size of LUVs used in calcein leakage assays, LUVs were measured via Dynamic Light Scattering after filtration. LUVs were prepared and filtered and DLS measurements of (A) DOPC (B) DOPC:DOPS or (C) DOPC:PI vesicles were performed. Size distribution graphs are representative of one preparation of LUVs.

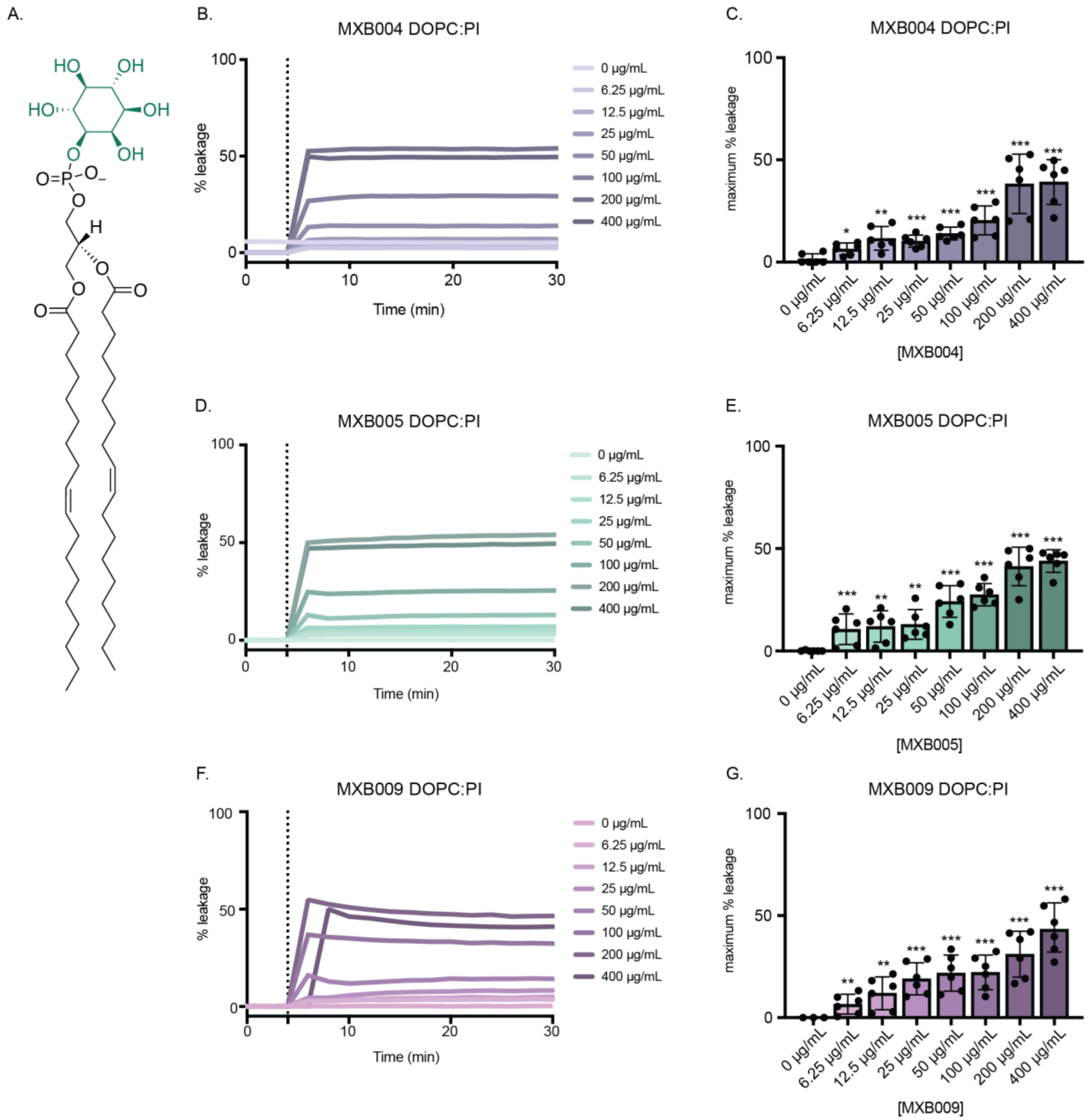

Figure S3. Antimicrobial peptoids induce membrane leakage in phosphatidylinositol containing vesicles. To determine if antimicrobial peptoids engage with other anionic phospholipids, vesicles containing phosphatidylcholine and phosphatidylinositol (*see phosphatidylinositol structure in A*) at a 70:30 molar ratio were generated. Background fluorescence of calcein-containing DOPC:PI Large Unilamellar Vesicles was measured for 4 minutes. At 4 minutes (B) MXB004 (D) MXB005 or (F) MXB009 was added at indicated concentrations. At 30 minutes, 10% Triton was added to achieve maximum fluorescence. Calcein release was normalized to the highest and lowest fluorescent values as percent leakage. Maximum fluorescence of (C) MXB004 (E) MXB005 or (G) MXB009 was calculated. Time courses are representative of one experiment and maximum percent leakage values are representative of three individual preparations of Large Unilamellar Vesicles with technical duplicates. *p<0.05, **p<0.01, ***p<0.001 by Student's T-test comparing treatment to untreated conditions (N≥3). Error bars represent on standard error of the mean.

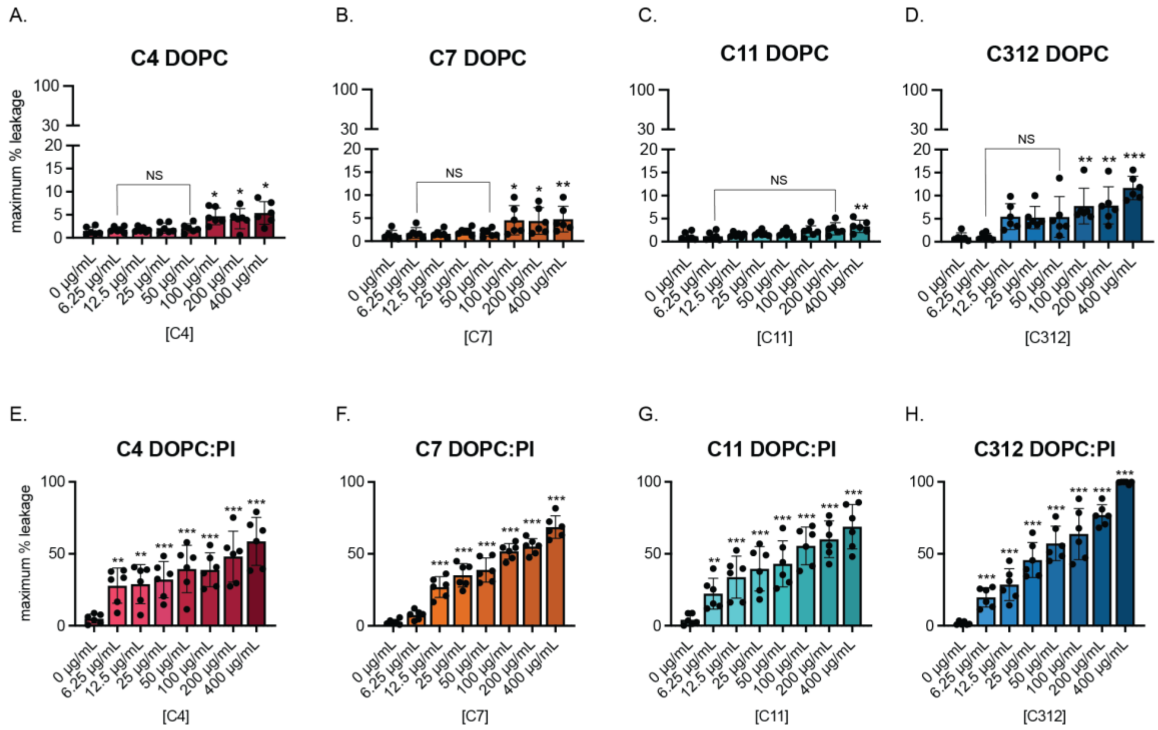

Figure S4. Liposome leakage profiles of macrocyclic peptoids. To determine how macrocyclic peptoids engage with different lipid vesicles, liposomes containing either phosphatidylcholine or both phosphatidylcholine and phosphatidylinositol were generated with encapsulated calcein. Background fluorescence of DOPC or DOPC:PI vesicles was measured up until 4 minutes. At 4 minutes, (A) C4 (B) C7 (C) C11 or (D) C312 was added at indicated concentrations. At 30 minutes, 10% Triton was added to liposomes to achieve maximum fluorescence. Calcein release was normalized to the maximum fluorescent values relative to the lowest measured fluorescent values as maximum percent leakage. Maximum percent leakage was also measured upon peptoid treatment in vesicles containing both DOPC and PI (E-H). Maximum percent leakage values are representative of three individual preparations of Large Unilamellar Vesicles with technical duplicates. NS not significant, *$p<0.05$, **$p<0.01$, ***$p<0.0001$ by Student's T-test comparing treatment to untreated conditions ($N\geq3$). Error bars represent on standard error of the mean.
